# Toward Joint Acquisition-Annotation of Images with Egocentric Devices for a Lower-Cost Machine Learning Application to Apple Detection

**DOI:** 10.3390/s20154173

**Published:** 2020-07-27

**Authors:** Salma Samiei, Pejman Rasti, Paul Richard, Gilles Galopin, David Rousseau

**Affiliations:** 1 Laboratoire Angevin de Recherche en Ingénierie des Systèmes (LARIS), Université d’Angers, 62 Avenue Notre Dame du Lac, 49035 Angers, France; salma.samiei@univ-angers.fr (S.S.); pejman.rasti@univ-angers.fr (P.R.); paul.richard@univ-angers.fr (P.R.); 2 UMR 1345 Institut de Recherche en Horticulture et Semences (IRHS), INRAe, 42 Rue Georges Morel, 49071 Beaucouzé, France; gilles.galopin@agrocampus-ouest.fr; 3 Department of Data Science, école D’ingénieur Informatique et Environnement (ESAIP), 49124 Angers, France

**Keywords:** egocentric vision, image annotation, apple detection, eye-tracking

## Abstract

Since most computer vision approaches are now driven by machine learning, the current bottleneck is the annotation of images. This time-consuming task is usually performed manually after the acquisition of images. In this article, we assess the value of various egocentric vision approaches in regard to performing joint acquisition and automatic image annotation rather than the conventional two-step process of acquisition followed by manual annotation. This approach is illustrated with apple detection in challenging field conditions. We demonstrate the possibility of high performance in automatic apple segmentation (Dice 0.85), apple counting (88 percent of probability of good detection, and 0.09 true-negative rate), and apple localization (a shift error of fewer than 3 pixels) with eye-tracking systems. This is obtained by simply applying the areas of interest captured by the egocentric devices to standard, non-supervised image segmentation. We especially stress the importance in terms of time of using such eye-tracking devices on head-mounted systems to jointly perform image acquisition and automatic annotation. A gain of time of over 10-fold by comparison with classical image acquisition followed by manual image annotation is demonstrated.

## 1. Introduction

In the era of machine learning-driven image processing, unequaled performances are accessible with advanced algorithms, such as deep learning, which are highly used in computer vision for agriculture and plant phenotyping [1]. The bottleneck is no more the design of algorithms than the annotation of the images to be processed. When performed manually, this annotation can be very time consuming, and therefore very costly. Consequently, it is useful to investigate all possibilities to accelerate this process. Annotation time can be reduced via multiple approaches, which have all started to be investigated in the domain of bioimaging and especially plant imaging [2,3,4,5,6,7,8,9]. First, (i) annotation time can be reduced by parallelizing the task via online platforms [5]. Additionally, (ii) it can be reduced by using shallow machine learning algorithms that automatically select the most critical images or parts of the images to be annotated via active learning [4]. Transferring segmentation models (iii) learned over available datasets can significantly reduce the need for annotated data [10]. Another approach to reducing annotation time (iv) is to do the training on synthetic datasets that are automatically annotated [2,3,6,7,9,11]. At last, (v) annotation time can be reduced via the use of ergonomic tools, which enable human annotators to accelerate the process without loss of annotation quality [8]. In this article, we contribute to the latter approach (v) to reduce annotation time. We introduce a novel use of egocentric devices in computer vision for plant phenotyping and assess their value to speed up image annotation.

The term “egocentric device” is used to designate all wearable imaging systems that record images from the first-person perspective. Images captured from egocentric devices are possibly of high value, since their field of view benefits from the attention of the person who wears the device and who is in charge of the targeted task to be done on the images. Reducing the field of view to a part of specific interest may reduce the complexity of the inspected scene and thus help the automatic processing of the acquired images. This is expected to be especially useful in complex scenes, such as those found outdoors in agriculture and phenotyping in the fields. Additionally, some egocentric devices, namely, head-mounted eye-trackers, can even include the capture of the ocular position of the annotator during the recording of the videos. This would, in theory, open up the possibility to annotate images directly, whereas acquisition and annotation are usually two separate steps. Such use of egocentric devices opens up the possibility to conduct these steps jointly and hence reduce annotation time. However, eye-trackers can never be perfectly calibrated, and their practical value in terms of both performance and time is still to be assessed in order to speed up annotation. That is what we propose here.

For the first application of egocentric devices to accelerate annotation, we considered as a proof of concept, a standard problem in computer vision for plant phenotyping. We chose the detection, i.e., segmentation, counting, and localization of apples in color images. This task has been addressed in many ways, including recently, with deep learning. This canonical problem is challenging for computer vision, since it includes self-occlusion of multiple instances, occlusion by the shoots of the apple trees, the variation of illumination, clutter from the self-similar background, variety in sizes and colors of fruits, etc. Additionally, this computer vision problem is significant for various agricultural applications, such as the design of automatic harvesters, automatic estimation of the fruit pack out, and variety testing. Most state-of-the-art methods developed for apple detection are currently working with supervised learning. Such methods require annotated images of apples to be efficient. In this article, we demonstrate how the use of egocentric devices can accelerate the annotation of apples in images. This acceleration in image annotation, illustrated here with apples, is of high value since it could benefit from reducing the annotation cost of any supervised learning segmentation method.

**Figure 1:**
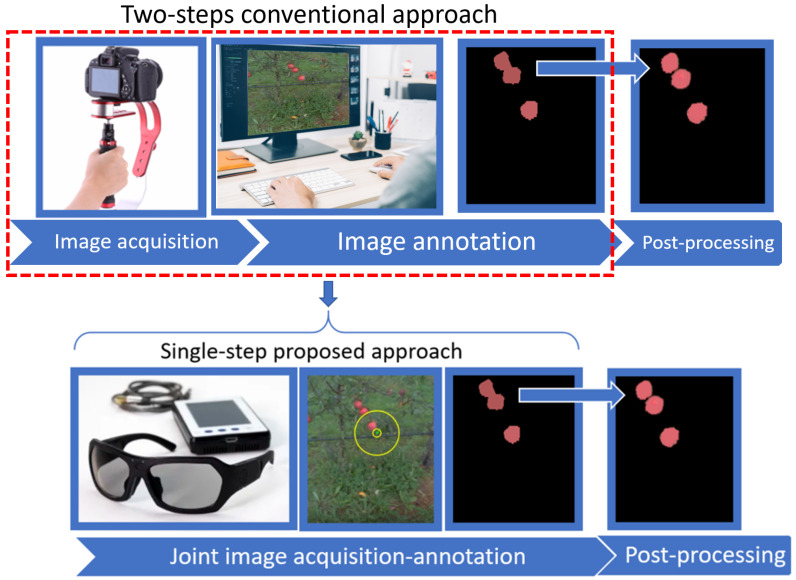
Visual abstract of the article. The red dotted-line encapsulates the conventional two steps of the acquisition and annotation process. We jointly perform image acquisition and image annotation by the use of a head-mounted egocentric device, which simultaneously captures images and the gaze of the person who wears the device and reaps benefits from both factors to annotate images automatically. It is to be noted that the post-processing step to separate touching annotated objects is not included here. It remains a step necessary in the conventional two-step approach and our proposed single-step approach.

A visual abstract of the proposed original approach for a joint image acquisition-annotation process is illustrated with apple detection in Figure 1. For comparison, the conventional approach is also depicted in Figure 1 wherein a handy camera is used to acquire images, and after image transfer to a computer, images are manually annotated. We propose a single-step approach where hands-free, head-mounted cameras with embedded computational resources are jointly acquiring and annotating images. The article is organized as follows. After positioning our work with the most related work (Section 2), we present (Section 3) the egocentric devices used, the acquisition protocol, and the dataset created for this study. A classical algorithm adapted from the literature is described, as we use it to detect apples in color images (Section 4). The same algorithm is then applied to compare five different computational strategies, specially designed for this study, to reap benefits from egocentric vision (Section 5). We finally conclude on the best practice identified via this comparison.

## 2. Related Work

Egocentric (first-person) vision is a relatively new research topic in the field of computer vision which is increasingly attracting interest for understanding human activities [12,13,14,15], object detection [16,17], creation of models of the environment with different levels of precision [18,19], perception of social activity [20], user–machine interactions [21], driving assistance [22], and medical applications [23,24,25]. There are different types of egocentric systems, such as smart glasses, action cameras, and eye-trackers. Based on the processing capabilities, embedded sensors, such as the one used in this article, are now more and more utilized in conjunction with egocentric video analysis [21]. Features such as hand appearance and head motion give essential cues about the attention, behavior, and goals of the viewer [26,27,28,29]. In our case, we also used the fact that, usually, in egocentric vision, salient objects of interest tend to occur at the center of the image, since they attract the attention of the viewer [16,30]. In this article, we primarily used an eye-tracking system for egocentric vision to speed up image annotation. The use of eye-tracker to speed up image annotation has been proven useful for annotation with a screen-based system in [8,31,32]. Those studies demonstrated a possible gain of time for annotation of 30-fold (approximately) by comparison with manual annotation. Here, we use, for the first time to the best of our knowledge, an embedded eye-tracking system in the form of glasses (see Figure 1) to jointly conduct image acquisition and annotation and thus extend the results of [8,31,32]. Embedded eye-tracking systems are known to be less accurate than screen-based eye-tracking systems because they can move slightly on the head of the observer during acquisition. However, embedded eye-tracking systems open the door for an accelerated procedure with joint acquisition and annotation, as illustrated in Figure 1. In this article we will compare the performances in terms of accuracy of apple detection and annotation time of both screen-based eye-tracking systems and embedded eye-tracking systems for image annotation.

Object detection in agricultural conditions has been investigated with a large panel of computer vision approaches [33,34,35,36,37,38,39,40,41,42,43,44,45]. In the early works, such as [33], methods were handcrafted both from the hardware side and the software side. Nowadays, it is more common practice to use standard RGB cameras, and base the detection of apples on supervised machine learning methods learned end-to-end via deep learning, as in [44,45]. Such modern methods, neural network-based, show high performances but require large amounts of annotated images. Manual pixel-wise annotation is, in general, a time-consuming operation, taking approximately 1.5 h per 100 images (308×202 pixels). In practice, apple detection is also challenging because of illumination conditions [46,47,48]. In this article, we will not provide a novel method to detect apples automatically. Instead, we will investigate the possibility of performing acquisition and annotation of apples in an orchard environment simultaneously by using head-mounted egocentric devices. Indeed, while there has been significant recent interest in fruit detection, segmentation, and counting in orchard environments, the cost of providing a unified annotated dataset of the fruit on trees makes it the bottleneck in the state-of-the-art literature [49].

The head-mounted egocentric camera provides areas of interest located in the vicinity of the targeted objects in the scene. Therefore, these areas of interest are less accurate than if a manual annotator was pointing at the object with a mouse. We propose in this article to test a standard image segmentation approach to detect the targeted object in the areas of interest provided by the head-mounted egocentric camera. As a consequence, the work relates to the literature on weakly or semi-supervised learning [50] with inexact supervision; that is, the training data are given with labels that are not as exact as desired. Different semi-supervised learning models have been introduced, such as iterative learning (self-training), generative models, graph-based methods, and vector-based techniques [51,52]. The color-based clustering technique for apple detection by using Gaussian Mixture Models was explained in [53]. In this approach, the SLIC superpixel was applied to the input image using the LAB color space. The superpixel’s results were clustered into approximately 25 color classes. Finally, based on the KL-divergence between Gaussian Mixtures, each superpixel was classified into an apple or background [54], from hand-labeled classes. Our objective was not to design a novel semi-supervised algorithm. Instead, we revisited existing standard methods based on superpixels and assessed the value of the areas of interest extracted by the head-mounted egocentric camera for a given task of object detection.

## 3. Material and Method

### 3.1. Egocentric Vision Device

The egocentric imaging system used was VPS-16 head-mounted eye-tracking glasses equipped with stereoscopic cameras in the nose bridge, a front camera with a diagonal coverage of 88 degrees, and an audio microphone sampling at 10 kHz. The front camera was calibrated with the eye-tracker before acquisition. The visual task defined to the wearer was to find apples on the targeted trees. The acquisition time was nearly 90 s for the whole dataset (calibration time included). This acquisition time is quite similar to the time required with a digital camera fixed on a tripod or hand-held, the former of which would need to be located in different positions to cover all apples located on a tree. The distance of the viewer and the tree was set approximately to one and a half meters. The viewer was counting the number of apples as evidence of the ground-truth, which was recorded via the audio microphone. Fixation points were recorded by the eye-tracker to investigate how they could serve to automatically annotate apples on the trees.

### 3.2. Dataset

**Figure 2:**
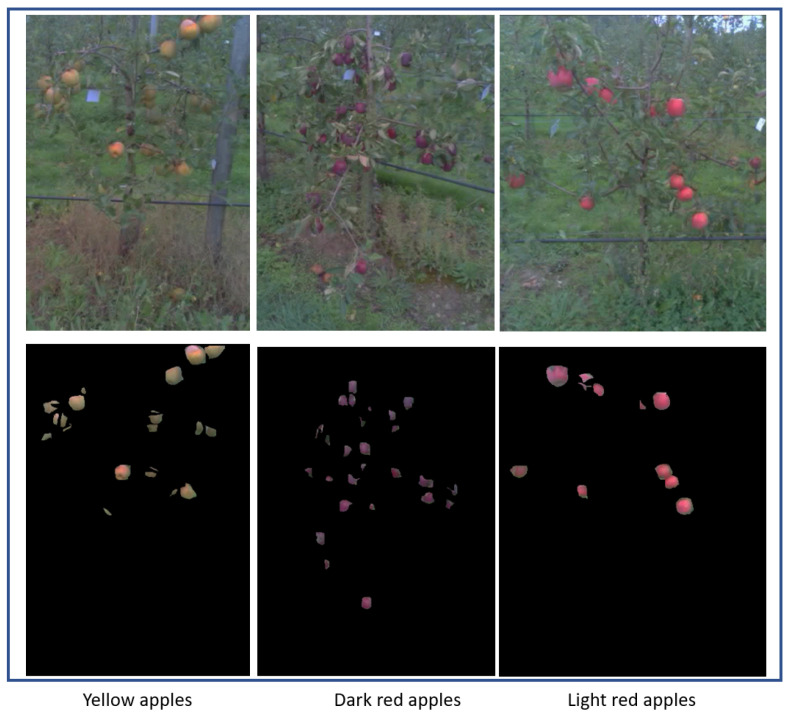
Example of RGB images of apple trees from our dataset and the corresponding ground-truth (manually annotated).

With the sensor described in the previous subsection, we generated a new dataset of 10 videos (25 fps) from 10 various apple trees in the orchard environment captured by the egocentric head-mounted glasses’ eye-tracker. The total number of extracted images from the entire dataset was 24,618 (frames). A fundamental parameter of eye-tracking analysis depends on the definition of the fixation and the algorithm used to separate fixation from saccades [55]. Fixation refers to a person’s point-of-focus as they look at a stationary target in a visual field. Although the mean duration of a single fixation may depend on the nature of the task [56], numerous studies have been done to measure the average duration for a single fixation [56,57,58,59,60,61,62,63,64,65]. The mean fixation duration for visual search is 275 ms, and for tasks that require hand-eye coordination, such as typing, the mean fixation can be 400 ms [56]. Among our dataset, the number of frames which received gazing of at least 275 ms was 419. The acquisitions were made on two days at midday with different weather conditions at the orchard of INRAE Angers, France. No difference was found in the results of the data coming from the two days. This dataset includes a variety of apple colors together with apple and foliage density, which are representative of the dataset found in the literature for apple detection [66,67,68]. Due to the complexity of each orchard tree, the illumination, and the environment itself, different natural colors were found in the images, including various shades of green, red, yellow, brown, or gray for the appearance of foliage, grass, apples, and tree trunks. Ground-truth was created by manual annotation of the raw color images at approximately 54 s per image by using the Image Segmenter application in Matlab 2017a. A sample of raw color images from different apple trees and their corresponding manual ground-truth are illustrated in Figure 2. For the whole dataset, which consists in 419 images, it roughly took 6 h to manually annotate all images. These manual annotations were generated for evaluation of the accuracy of the egocentric vision methods presented in the next section.

## 4. Image Processing Pipeline

**Figure 3:**
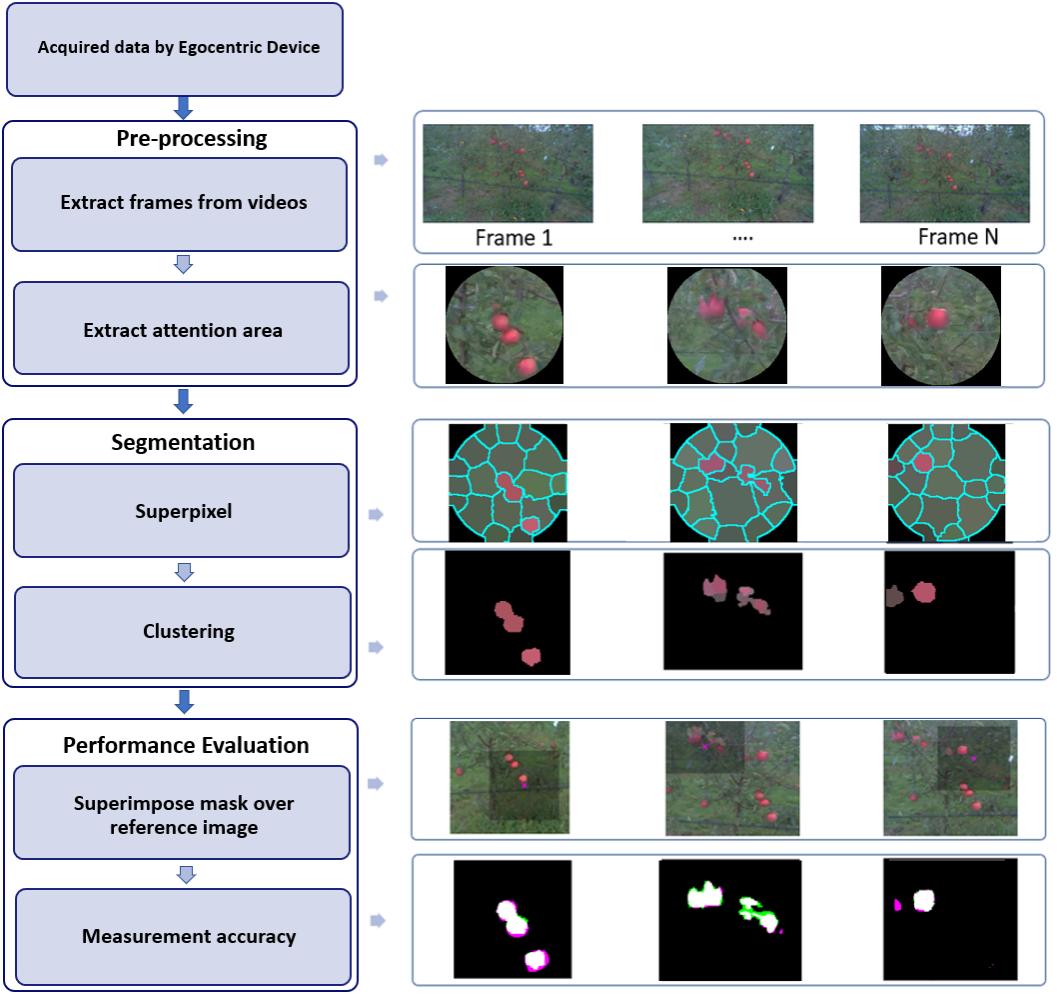
The three-step image processing pipeline proposed to automatically segment apples from the attention areas captured with egocentric devices.

In this section, we present the image processing pipeline developed to automatically annotate apples from the attention areas captured with egocentric vision. A global view of this pipeline is depicted in Figure 3 and includes three main steps: image pre-processing, segmentation, and performance evaluation.

The pre-processing started with the extraction of the frames with a resolution of 960×544 pixels from recorded videos. Next, an attention area was extracted from each frame based on egocentric priors. The extraction of this attention area constitutes the main contribution of the article. Several strategies have been tested and are presented in the next section. The pre-processed images were then segmented with a standard approach for apple detection similar to the one presented in [49,53,69,70,71]. A classical superpixel technique (SLIC) [72] was applied followed by a simple non-supervised clustering technique, *K*-means [73], to select superpixels corresponding to apples. To keep the size of superpixel independent of the size of the attention area, we defined the number of superpixels as the ratio of

(1) $$N=\frac{A}{S},$$

where *A* represents the size of the attention area, and *S* the size of an average apple, which is equal to 900 pixels in our dataset.

**Figure 4:**
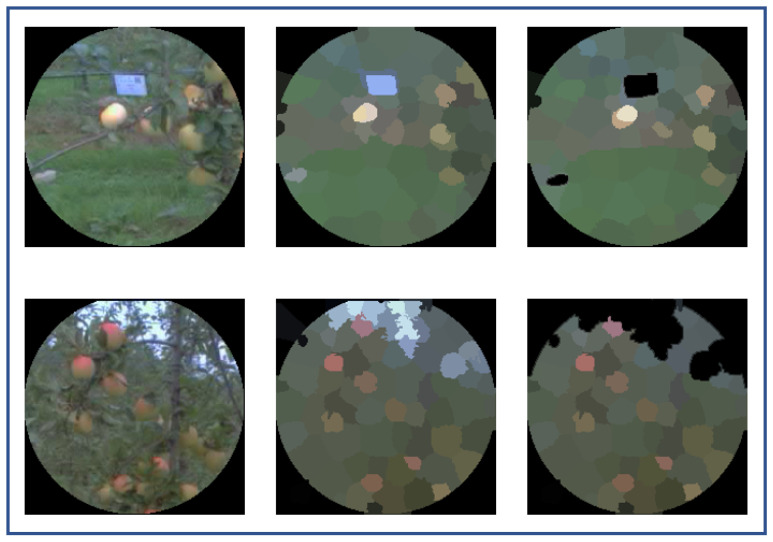
Color thresholding to remove blueish color belonging to the sky or blue tree-labels on superpixel segmented attention areas. Each row represents from left to right: the attention area, the superpixel segmented attention area, and the thresholded one, respectively.

To simplify the images, the tree-labels (blue in our case) and sky parts were removed by applying color thresholding (optimized on a small dataset) in the RGB color domain on the superpixel segmented attention areas, as shown in Figure 4. The number of cluster *K* was found optimal for K=2 and was applied to feature space composed of (R,G,B,H,S) respectively for red, green, brightness, hue, and saturation from each superpixel. The cluster with the smaller size was considered as the apple cluster based on the assumption that the background occupied the largest area in the attention area. Because blue parts were withdrawn and no green apples were present, the optimal value of K=2 was reasonable for our use-case of apple detection in the orchard. Indeed, the local complexities in attention areas extracted from the egocentric devices were limited to objects on a background with a contrast of color. For other use-cases, where local contrast between the object and background could depend on other features (size, texture, shape, etc.), it would be necessary to adapt this segmentation.

Finally, the segmented apples were superimposed over the original image for qualitative assessment and localization, and compared with the manual binary ground-truth to compute the segmentation accuracy via the Dice Dc(X,Y) and Jaccard index J(X,Y) given by

(2) $$Dc\left(X,Y\right)=\frac{2*\left|X\cap Y\right|}{\left|X\right|+\left|Y\right|},$$

(3) $$J\left(X,Y\right)=\frac{\left|X\cap Y\right|}{\left|X\cup Y\right|},$$

where *X* and *Y* represent the segmented image and the ground-truth respectively.

In addition to the segmentation of apples, counting and localization were also computed in the following way. For object counting, we counted the number of connected components among detected objects which shared sufficient overlaps with ground-truth. An empirical threshold of 75 percent was chosen for the overlap. The probability of good detection was computed as

(4) $$PD=\frac{TP}{TP+FN}\;,$$

with TP number of true-positive objects and FN number of false-negative objects. We also computed the probability of true-negative rate as

(5) $$TNR=\frac{TN}{TN+FP}\;,$$

with TN number of true-negative objects and FP number of false-positive objects.

In localization, the Euclidean distance between the centroid $x_{i}$ of detected objects $X_{i}$ and the centroid $y_{j}$ of objects $Y_{i}$ with a maximum intersection with ground-truth was computed as

(6) $$d\left(x_{i},y_{j}\right)=\sqrt{{\left(u_{x_{i}}-v_{y_{j}}\right)}^{2}+{\left(u_{x_{i}}-v_{y_{j}}\right)}^{2}}\;,$$

with *u* and *v*, which stand for Cartesian coordinates in the images and

(7) $$j=\arg\max_{j_{0}}\left|X_{i}\cap Y_{j_{0}}\right|\;.$$

The average distance

(8) $$d=\frac{1}{N}\sum_{i=1}^{N}d\left(x_{i},y_{j}\right),$$

was computed over all detected objects sharing sufficient overlap with ground-truth. Here again, a threshold of 75 percent of overlap was chosen. Distance *d* represents the average shift error of localization of apples with an egocentric device from manual ground-truth.

## 5. Strategies for Extracting Attention Area

In the following we mention different approaches for extracting attention area either using eye-tracking or not.

### 5.1. Attention Area from Eye-Tracking

In this section, we present strategies that we developed to extract attention areas from the eye-tracking devices to perform joint acquisition-annotation after passing these areas to the image processing pipeline of the previous section.

#### 5.1.1. Selection by Eye-Tracking Glasses

The first approach extracted attention areas via the viewer fixation computed from the egocentric eye-tracking glasses. In order to fix a threshold, a gazing position was recorded when the same fixation position was observed during an interval of 6 frames, as calculated by

**Figure 5:**
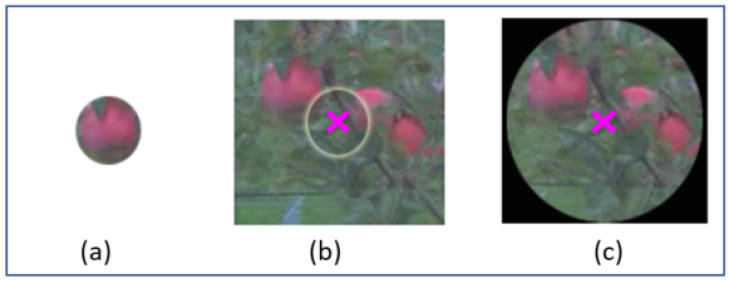
Construction of attention areas. (**a**) The average diameter of an average apple is 30 pixels in our dataset; (**b**) a cross indicates the center of the gaze of the annotator. There is a shift error from the apple of (**a**). The maximum distance of the gazing point with the center of the closest object was found at 169 pixels. (**c**) Chosen attention area with a size of 180×180 pixels.

**Figure 6:**
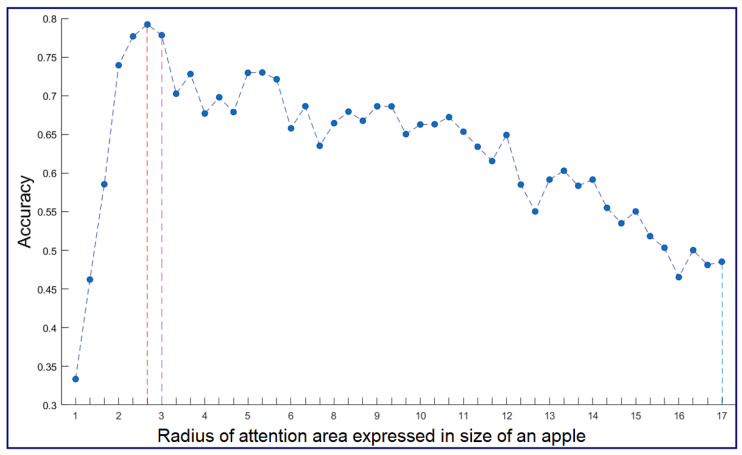
Apple segmentation accuracy as a function of the radius of attention area expressed in the size of apples taken as 30 pixels. Maximum accuracy achieved when the radius size of the attention map is equal to 80 (160×160 pixels) corresponding to the red dotted line. The purple dotted line corresponds to the maximum gaze shift error of (169 pixels) between eye-tracker and ground-truth when computed on the whole dataset.

(9) fi=Fps×fd,

where fi is the frame interval, Fps=25 is the number of frames per second, and fd is the average fixation duration, which was set as 275 ms. Despite careful calibration before the acquisition, small shift errors of alignment between the front camera of the device and the gazing point of the viewer can occur. Therefore, we extended the attention area around each gazing position with a given radius to compensate for the remaining small shift error of calibration of the eye-tracker. An illustration of the creation of an attention area around a fixation point is provided in Figure 5. A systematic analysis of the evolution of the average segmentation accuracy as a function of the radius of the attention area around each gazing position was undertaken. It is shown in Figure 6 and demonstrates a non-monotonic evolution culminating at a value corresponding to triple the size of an average apple size in our dataset. Consistently, this optimal value was also found to be very close to the maximum shift error of calibration of the eye-tracker found in the whole dataset. For attention areas that are too small, due to the shift error, apples can be missed. For overly large attention areas, due to the complexity of the scene, the segmentation process fails to detect all apples correctly in the area.

#### 5.1.2. Selection by Screen-Based Eye-Tracking

For comparison with the attention area created with the egocentric eye-tracker directly acquired in the orchard, we also generated an attention map from the gazing point recorded with a screen-based eye-tracker. Of course, this approach is less interesting for gain of time than the previous one with the head-mounted eye-tracker, since it does not allow a joint acquisition annotation. However, desktop eye-trackers are more accurate than head-mounted ones and thus are expected to constitute a reference serving as an upper bound in terms of quality of annotation with ego-centric vision. The experiment was performed on a screen with a resolution of 1920×1080 pixels while the eye movements of the viewer were recorded with an SMI binocular remote eye-tracker [74]. In this approach, for each apple tree, we peaked out one frame, which included all the apples.

The annotation protocol was the same as in the previous method. Each image was displayed to the viewer, who was asked to find the apples on the trees. The locations of the fixations of the viewer were recorded at 60 Hz. For a fair comparison, the attention area diameter around each recorded fixation was taken at the optimal value found for the eye-tracking systems embedded in glasses.

**Figure 7:**
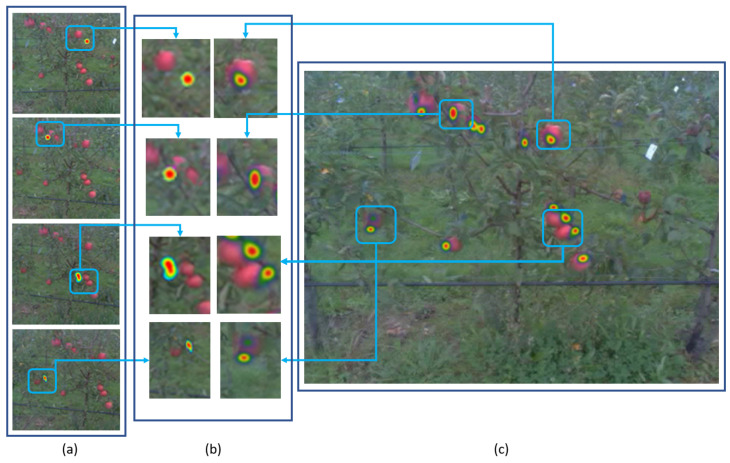
Heatmap visualization of the attention of the viewer captured by the head-mounted (glasses) eye-tracker (**a**) versus the screen-based eye-tracker (**c**). (**b**) Comparison of the heatmap generated by the glasses eye-tracker (left) vs. the heatmap generated by the screen-based eye-tracker (right).

A comparison of the accuracy of the screen-based eye-tracking recording and the recording with eye-tracking embedded in glasses was conducted. Figure 7 shows that in the form of heatmap visualization of the attention of the viewer. The precision and accuracy of the produced gaze points with the screen-based eye-tracker were found to be higher than when using the head-mounted eye-tracker. The average shift error of Equation (8) was found to be 125 pixels less with the screen-based eye-tracker than with the head-mounted eye-tracker.

### 5.2. Attention Area without Eye-Tracking

Other strategies were developed to extract attention areas for comparison with performances obtained with eye-tracking systems.

#### 5.2.1. Full-Frame

In this approach, the attention map was considered as the full-frame recorded by the camera. Thus, in Figure 3, instead of a small patch of the entire original image, the full original image was directly transmitted to the superpixel segmentation. Such a choice assumes that the camera field of view is already a focus of the overall field of interest for the human annotator in charge of detecting apples.

#### 5.2.2. Egocentric Prior

In this approach, we assumed, as is often done in egocentric vision [16], that the attention of the viewer was focused at the center of the frame. Therefore, we selected the attention area as a disk positioned at the center of the image with the size of 180×180 pixels for a fair comparison with the other approaches developed for eye-trackers.

#### 5.2.3. Saliency Map

As the last method to compute an attention area, we turned toward a computational approach in charge of numerically identifying areas of interest. Such a concept has been developed in the computer vision literature under the name of the saliency map. Saliency acts as a local filter that enhances regions of the image which stand out relative to their adjacent parts in terms of orientation and/or gray level and/or color contrast [75]. Introduced in [76], saliency was inspired by the mechanisms of human visual attention and the fixation behavior of the observer. There are numerous computational models for salient object detection. In this study, for illustration and without any claim of optimality, we used the algorithm proposed by [77], which computes saliency map in images using low-level features and was proposed with codes included for reproducible science. Saliency maps were thresholded to binary masks following the fixed threshold procedure described in [77]. Each connected component of the binary saliency map served to produce an attention area. For a fair comparison with the other approaches, attention areas of size 180×180 pixels were chosen.

## 6. Results and Discussion

We are now ready to compare the results of the different approaches proposed for apple detection by extracting attention areas through egocentric vision in the perspective of a joint acquisition-annotation process. As shown in Table 1, we assessed the image annotation quality by the same image segmentation pipeline of Section 4 (depicted in Figure 3). Comparison is provided between the five different approaches presented in Section 5 for the extraction of attention areas from egocentric devices. In terms of segmentation, accuracy was estimated by the Dice Equation (2) and Jaccard Equation (3) indexes. The probability of good detection indicates the true counted apples computed by Equation (4). The true-negative rate Equation (5) represents the proportion of actual negatives that are correctly identified. The next column in Table 1 specifies the error of localization of detected apples computed by Equation (8). Time is the approximate consumed execution time (automatic annotation) acquired from each approach of the whole dataset. Finally, the time gain indicates the ratio of manual annotation time over the consumed execution time obtained from each automatic annotation approach. All these experimental results correspond to an average of over 10 different trees available in the dataset.

**Figure 8:**
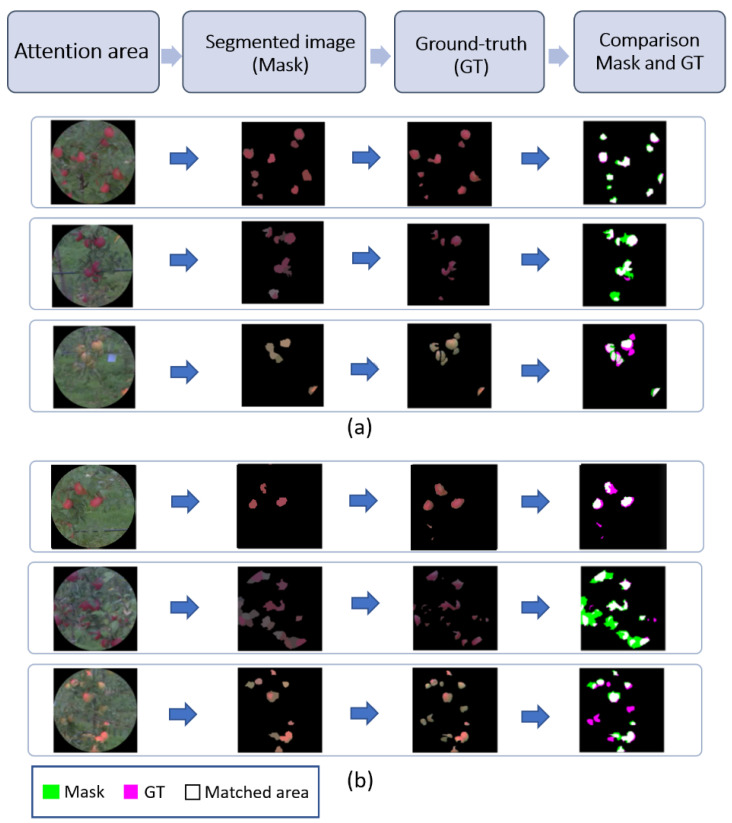
Qualitative assessment of results. From left to right, an example of the attention area captured by eye-tracking, automatic annotation obtained from the proposed image processing pipeline of Figure 3, ground-truth manually recorded, and comparison of manual ground-truth and automatic segmentation. (**a**) Examples of good performance; (**b**) Some challenging conditions wherein more errors were found (missed detection, false detection).

The best average performances (highlighted in bold in Table 1) in terms of segmentation accuracy of apples were obtained with the eye-tracking-based methods. Challenging images and resulting annotations with eye-tracking-based methods are provided in Figure 8 for qualitative assessment. Overall, the screen-based eye-tracker provided the best result but only slightly above the one obtained from the glasses eye-tracker. This embedded glasses eye-tracker, despite its substantial shift errors, had a high value since it enabled joint image acquisition and annotation. The saliency approach provided a result close to the one obtained with the baseline method (full-frame). This could certainly be improved with a systematic benchmark of other saliency methods of the literature. However, a fundamental reason for the failure of the saliency approach, which would be common to all generic saliency maps, is that saliency is, so to say, attracted by contrasting objects which may not be apples (for example, stems, leaves, items in the background, a data matrix positioned in the field to identify trees). As a consequence, saliency creates many true-negatives in attention areas since the task of detecting apples does not specifically drive it. In contrast, human attention focuses on the apple as captured by eye-tracking systems.

Interestingly these results were consistent for the three tasks assessed: segmentation, counting, and localization. This demonstrates the robustness of the interest of eye-tracker devices for annotation. Eye-tracking systems, such as the two used in this study, can be considered as expensive devices (typically between 10,000 and 20,000 euros currently). It is interesting to see that the egocentric prior approach gave the third-best performance, and this could be accessible with any camera embedded on glasses (for 10 to 100 euros).

The values of the obtained results in terms of segmentation, counting, and localization were also assessed in terms of timing. As expressed in Section 3.1, acquisition time with an egocentric device is comparable with acquisition time with any standard camera. Therefore gains of time were compared regarding the annotation time only. This timing is provided in the last column of Table 1 for automatic annotation based on the image processing pipeline applied to extracted attention areas. Without any surprise, the full-frame approach, which requires no computation of attention map, is the fastest method. The second most rapid methods are the egocentric prior and glasses eye-tracker. The screen-based eye-tracker method, which gave the best performance in terms of apple detection, came with the slowest timing. However, these timings for automated annotation are to be compared with the timing requested by a human annotator to manually annotate all apples in the dataset. The estimated timing was 6 h for the 419 frames. The gain of time for all methods is presented in Table 1. Saliency, as presented here, could be criticized since many other variants of the saliency map could be tested and possibly provide better results. In terms of timing, however, we believe the performances are realistic, and it was worth mentioning them here. All in all, the glasses eye-tracker method appears to be a good trade-off between speed and annotation performance (as summarized in Table 2). For this head-mounted device, the gain in performance was about 11 times, which is smaller than what was found in the closest related work with desktop eye-trackers for object detection [8,31,32]. This difference may come from the fact that in this literature, the tasks targeted were relatively more straightforward and required less post-processing. Optimization of the code could thus increase the gain in time. We are currently investigating all those perspectives.

## 7. Conclusions

We have assessed the value of egocentric imaging devices to jointly perform acquisition and automatic image annotation. This was illustrated with apple detection in orchards, which is known to be a challenging task for computer vision applied to phenotyping or agriculture. Despite shift errors in the calibration of egocentric imaging devices, the performance of the detection of apples from the gazed recorded areas was found to be very close to the one obtained from the manual annotation. The compensation for these shift errors was obtained by applying a standard non supervised segmentation algorithm only applied in attention areas centered on the gazing positions captured by the egocentric devices. Specific interest was shown for head-mounted eye-tracking systems with an estimated gain of time in comparison with manual annotation of 11 times with non-GPU-accelerated software.

This first use of egocentric vision to speed up image annotation opens up interesting perspectives, especially in plant phenotyping. The task here was focused on apples, but the approach is in fact generic. Thus, it would be interesting to extend the applicability to other phenotyping items of interest. The non-supervised image segmentation algorithm applied in gazed areas was purposely chosen simply in this article to demonstrate the value of the eye-tracking device. It is interesting to notice that performances obtained with this simple algorithm were already interesting quantitatively and qualitatively. The literature of non-supervised image segmentation with superpixels is huge [78,79], and it would be interesting to revisit more exhaustively this literature for the segmentation of gazed areas. Specific attention could focus on the methods addressing the limitation of superpixels [80], also observed in this article, with "leakage" of boundaries in the vicinity of the targeted objects [81]. To remain on the topic of apples, this could include the determination of flowering stages or the detection of diseases. Additional technological services from egocentric vision could be tested to speed up annotation. For instance, this includes the use of sound recording, which could be coupled to automatic speech recognition for later fusion with information extracted from the captured images. The pilot study presented here is promising. For a tool to be used by technicians and engineers in the field, it would be necessary to implement an ergonomic version of the software to experiment on a large network of users the method developed to accelerate image annotation with egocentric devices. Validation of the quality of the annotation was performed at various levels, including location, object detection, and pixel-wise segmentation. Another stage of validation of the quality of the annotation would be to train a machine learning algorithm on the annotated images and compare the performance with the manually annotated data.

## Figures and Tables

**Table 1:** Performance of apple detection with the five approaches developed for automatic apple annotation in the attention area captured by the egocentric devices. Each column corresponds to an average over the 10 trees of the dataset. Dice and Jaccard assess in percentage the quality of segmentation via Equations (2) and (3); good prediction and true-negative rate assess in percentage the quality of object detection via Equations (4) and (5); and the shift error of Equation (8) assesses in pixels the quality of good localization. The time corresponds to the approximate execution time for automatic annotation for the whole dataset in seconds. Time gain indicates the ratio of manual annotation time (6 h) over automatic annotation time obtained from each approach. Time was measured on a windows machine with an Intel Xeon CPU and 32.0 GB RAM by Matlab 2017a.

Method (Section)	Dice	Jaccard	Good Detection	True-Negative Rate	Shift Error	Time (Second)	Time Gain
Full-Frame (Section 5.2.1)	0.24 ± 0.22	0.21 ± 0.16	0.31 ± 0.20	0.17 ± 0.72	174.11 ± 34	880	24
Glasses Eye-tracker (Section 5.1)	0.78± 0.08	0.64 ± 0.08	0.84 ± 0.16	0.09 ± 0.07	15.97 ± 11	1960	11
Screen-based Eye-tracker (Section 5.1.2)	0.85± 0.09	0.77 ± 0.13	0.88 ± 0.12	0.09 ± 0.13	2.37 ± 1.86	3240	6
Egocentric Prior (Section 5.2.2)	0.46 ± 0.36	0.38 ± 0.31	0.54 ± 0.39	0.28 ± 0.23	84.82 ± 7.25	1960	11
Saliency (Section 5.2.3)	0.27 ± 0.13	0.16 ± 0.08	0.42 ± 0.45	0.51 ± 0.17	7.21 ± 8.28	2358	9

**Table 2:** Qualitative summary of the five uses of egocentric devices compared in this study.

Method	Joint Acquisition Annotation	Fastest Execution Time	Best Annotation	Best Counting	Best Localization
Full-Frame	+	+	-	-	-
Glasses Eye-tracker	+	-	+	+	-
Screen-based Eye-tracker	-	-	+	+	+
Egocentric Prior	+	-	-	-	-
Saliency	+	-	-	-	+
